# Intronic deletions of *tva* receptor gene decrease the susceptibility to infection by avian sarcoma and leukosis virus subgroup A

**DOI:** 10.1038/srep09900

**Published:** 2015-04-15

**Authors:** Weiguo Chen, Yang Liu, Hongxing Li, Shuang Chang, Dingming Shu, Huanmin Zhang, Feng Chen, Qingmei Xie

**Affiliations:** 1 College of Animal Science, South China Agricultural University & Guangdong Provincial Key Lab of Agro-Animal Genomics and Molecular Breeding, Guangzhou, 510642, P. R. China; 2 College of Veterinary Medicine, Shandong Agricultural University, Taian, 271018, P. R. China; 3 Institute of Animal Science, Guangdong Academy of Agriculture Sciences, Guangzhou, 510640, P. R. China; 4 USDA, Agriculture Research Service, Avian Disease and Oncology Laboratory, East Lansing, MI, 48823, U.S.A; 5 Key Laboratory of Animal Health Aquaculture and Environmental Control, Guangdong, Guangzhou, 510642, P. R. China; 6 South China Collaborative Innovation Center for Poultry Disease Control and Product Safety, Guangzhou, 510642, P. R. China

## Abstract

The group of avian sarcoma and leukosis virus (ASLV) in chickens contains six highly related subgroups, A to E and J. Four genetic loci, *tva*, *tvb*, *tvc* and *tvj*, encode for corresponding receptors that determine the susceptibility to the ASLV subgroups. The prevalence of ASLV in hosts may have imposed strong selection pressure toward resistance to ASLV infection, and the resistant alleles in all four receptor genes have been identified. In this study, two new alleles of the *tva* receptor gene, *tva*^r5^ and *tva*^r6^, with similar intronic deletions were identified in Chinese commercial broilers. These natural mutations delete the deduced branch point signal within the first intron, disrupting mRNA splicing of the *tva* receptor gene and leading to the retention of intron 1 and introduction of premature TGA stop codons in both the longer and shorter *tva* isoforms. As a result, decreased susceptibility to subgroup A ASLV *in vitro* and *in vivo* was observed in the subsequent analysis. In addition, we identified two groups of heterozygous allele pairs which exhibited quantitative differences in host susceptibility to ASLV-A. This study demonstrated that defective splicing of the *tva* receptor gene can confer genetic resistance to ASLV subgroup A in the host.

Entry of retroviruses into the host cell represents one of the most important steps in the viral life cycle^1^. The process is mediated by the interaction of retroviral envelope glycoproteins with specific cell surface receptors^2^. This interaction, as well as subsequent phases of virus entry, requires a perfect match of envelope glycoproteins and receptors^3^,^4^. Despite the strict structural requirements for these interactions, hypervariability of retroviral glycoproteins can change the receptor usage and broaden the host range. Avian sarcoma and leukosis viruses (ASLVs) in chickens are a closely related group of retroviruses thought to have evolved from a common viral ancestor into six subgroups, A to E and J, which utilize four different cell surface receptors encoded by four genetic loci, *tva*, *tvb*, *tvc* and *tvj*^1^,^4^,^5^,^6^. The susceptibility of chicken cells to subgroup A ASLV is determined by the *tva* locus, which encodes a protein belonging to the family of low-density lipoprotein receptors^7^. The susceptibility to the subgroup B, D and E ASLVs are determined by the *tvb* locus, which encodes the tumor necrosis factor receptor-related protein^8^,^9^,^10^. The Tvc protein encoded by the *tvc* locus, closely related to the mammalian butyrophilins, serves as the receptor for subgroup C ASLV^11^. The receptor for subgroup J ASLV was identified as chicken Na^+^/H^+^ exchanger type 1 (chNHE1), encoded by the *tvj* locus^6^.

The complete resistance or decreased susceptibility of host cells to a particular ASLV subgroup can be caused by genetic variations of the *tva*, *tvb*, *tvc* or *tvj* locus, resulting in the complete lack of receptor protein expression or the expression of an aberrant protein not suitable as a viral receptor. Genetic variations that confer host resistance to infection by specific ASLVs, *tva*^r^, *tvb*^r^ and *tvc*^r^ alleles, have been identified in some inbred lines of White Leghorn chickens. The mutations found in the resistant alleles result in premature termination or a frameshift in the receptor-encoding loci^11^,^12^,^13^, decreased receptor expression and display^14^, and even single amino acid substitutions in the receptor gene sequence^13^,^15^. In addition, the absence of tryptophan 38 of the cell surface receptor abrogates binding of the subgroup J envelope glycoprotein to ASLV-J-resistant cells, which discriminates sensitive from resistant avian species^16^.

Although research on host genetic resistance to specific ASLVs has progressed in recent years, the current status of host resistance to infection by specific ASLVs in Chinese chickens is unknown. In order to identify additional resistance alleles of the *tva* receptor gene, we screened a panel of Chinese commercial broiler lines, which have undergone strict virus eradication management. Here, we characterized two alleles of the *tva* receptor gene, named *tva*^r5^ and *tva*^r6^, respectively, with similar intronic deletions encompassing the deduced branch point signal within the first intron and leading to defective splicing of *tva* mRNA. We also identified two groups of heterozygous allele pairs which exhibited discrepant susceptibility to subgroup A ASLV. To our knowledge, this study is the first to report genetic variations within the *tva* receptor gene that result in a quantitative effect on ASLV-A susceptibility and pathogenesis in Chinese chickens.

## Results

### Polymorphisms in the first intron of *tva* receptor gene and genotyping

**Figure 1:**
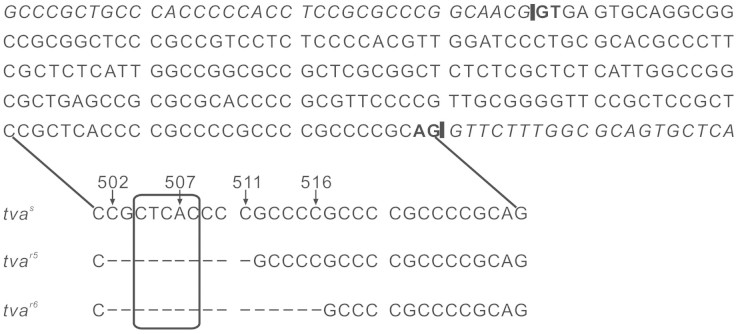
Alleles *tva*^r5^ and *tva*^r6^ in Chinese commercial broiler lines containing deletions in intron 1 of *tva* receptor gene. Partial genomic sequence showing intron 1 and junctions with exons 1 and 2 in the *tva* gene. The regions of the branch point with corresponding deletions in the *tva*^r5^ and *tva*^r6^ alleles are indicated below, and the deleted bases are represented by dashes. The deduced branch point signal is boxed, and the GT-AG intron termini are shown in bold. The underlined sequences indicate the putative alternative branch point signals. The intron-exon junctions are indicated by vertical bars, while exon sequences are indicated in italics. The nucleotide numbering based on the published *tva*^s^ allele genomic sequence (GenBank accession number: AY531262.1) is provided for reference.

To dissect genetic variations within the *tva* receptor gene in a panel of Chinese commercial broiler lines, the genomic region of the *tva* gene in each bird was amplified and sequenced. Four novel variants in the same region within the first intron of the *tva* gene were identified in the Chinese chickens surveyed. In addition to the *tva*^r3^ and *tva*^r4^ alleles^14^, sequencing revealed one new variant with a deletion of the sequence CGCTCACCCC (nucleotides 502 to 511) and another new variant with a deletion of the sequence CGCTCACCCCGCCCC (nucleotides 502 to 516) (Fig. 1). We designated these two novel variants as *tva*^r5^ and *tva*^r6^ alleles. These four variants in the *tva* receptor gene (*tva*^r3^, *tva*^r4^, *tva*^r5^ and *tva*^r6^) together may be considered a multiple allele, denoted here as *tva*^mut502–516^. The genotypic frequencies of the *tva*^mut502–516^ multiple allele within the *tva* receptor gene in the Chinese commercial broiler lines surveyed are presented in Table 1. All genotypes of the *tva*^mut502–516^ multiple allele were identified, including the *tva*^s/s^, *tva*^r3/r3^, *tva*^r4/r4^, *tva*^r5/r5^ and *tva*^r6/r6^ homozygotes, as well as two groups of heterozygous genotypes. One group of heterozygous genotypes is sensitive to ALSV-A, included *tva*^s/r3^, *tva*^s/r4^, *tva*^s/r5^ and *tva*^s/r6^, while the other group is resistant to ALSV-A, comprised of *tva*^r3/r4^, *tva*^r3/r5^, *tva*^r3/r6^, *tva*^r4/r5^, *tva*^r4/r6^ and *tva*^r5/r6^. This difference between the two groups of heterozygous genotypes was identified in the subsequent analysis. The genotypes and their frequencies in Chinese commercial broiler lines were obviously different (Table 1).

### Identification of novel *tva* receptor gene splicing variants

**Figure 2:**
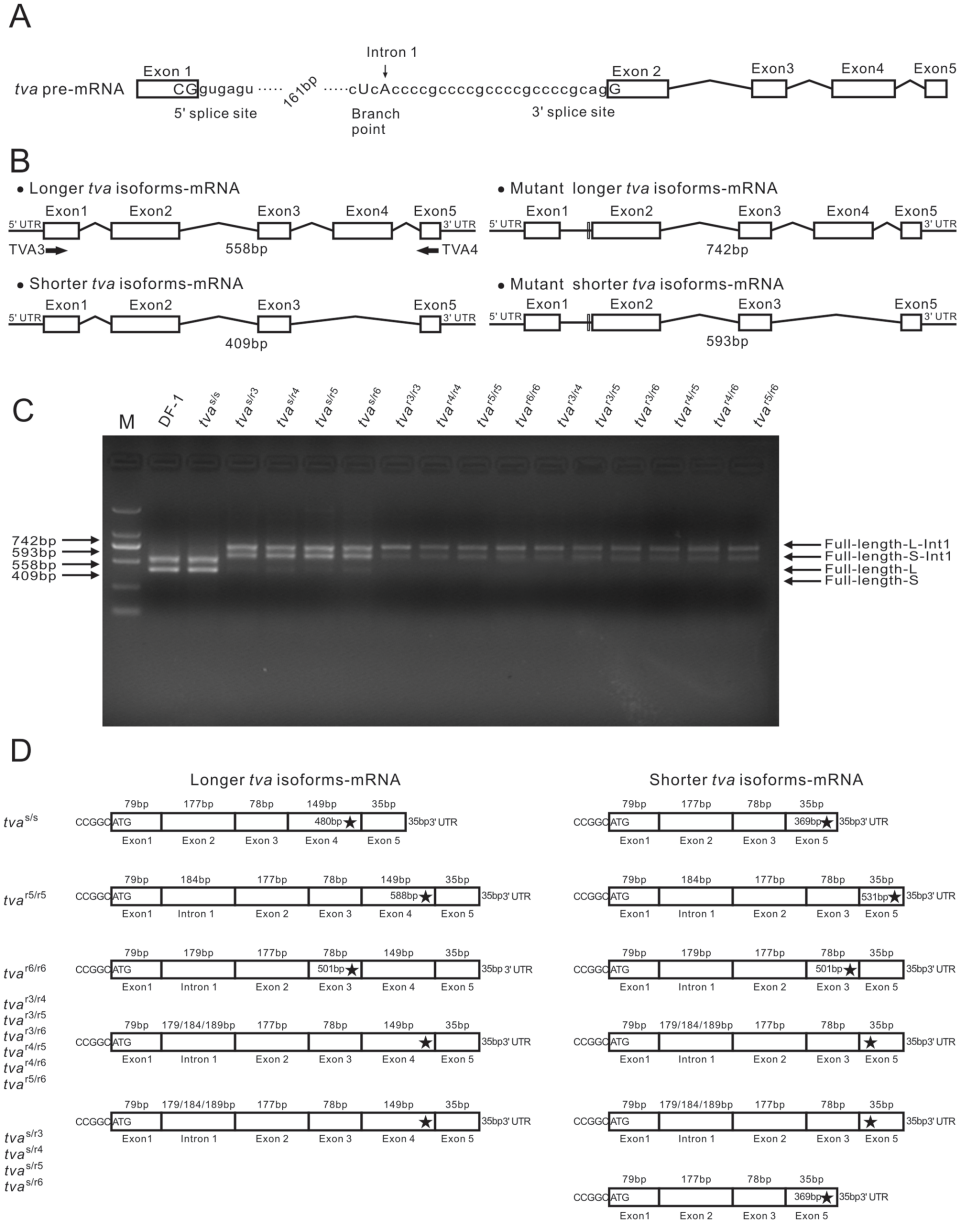
Deletion of internal intron 1 sequences affects splicing of *tva* receptor gene. (A) Schematic diagram of intron 1 in *tva* pre-mRNA showing the 5′ splice site, 3′ splice site and the branch point sequence (corresponding bases are indicated by dots). The adenosine residue which is required for the first cleavage-ligation step of the splicing reaction^18^ is marked by an arrow. (B) Schematic diagram of RT-PCR strategy. The use of PCR primers TVA3 and TVA4 generated easily discernible whole cDNA fragments that were amplified from the longer and shorter *tva* forms, respectively, as well as the longer and shorter *tva* forms with intron 1 retention. Sizes of diagnostic PCR products are indicated. Exons are drawn as boxes, retained introns are shown as black lines and spliced introns as diagonal lines. The vertical white bar indicates the position of the intronic deletion. (C) RT-PCR of RNA isolated from DF-I cells and samples from lives of defined origin. Lane 1–16 indicated the RT-PCR products from DF-I cells and the *tva*^s/s^, *tva*^s/r3^, *tva*^s/r4^, *tva*^s/r5^, *tva*^s/r6^, *tva*^r3/r3^, *tva*^r4/r4^, *tva*^r5/r5^, *tva*^r6/r6^, *tva*^r3/r4^, *tva*^r3/r5^, *tva*^r3/r6^, *tva*^r4/r5^, *tva*^r4/r6^ and *tva*^r5/r6^ samples from lives, respectively. Positions of full-length and intron 1 retention PCR products are indicated on the right, and sizes of diagnostic PCR products are indicated on the left. Spliced products from the full-length longer and shorter *tva* forms (Full-length-L and Full-length-S) migrated slightly faster than the corresponding RNAs of the mutants (Full-length-L-Int1 and Full-length-S-Int1). The gels have been run under the same experimental conditions, and the cropped gels are used. The full-length gel images are presented in the supplementary information. (D) Separate sequence analysis of PCR products revealed normal splicing of longer and shorter *tva* forms of *tva*^s/s^ homozygotes, while splicing of longer and shorter *tva* forms of *tva*^r5/r5^ and *tva*^r6/r6^ homozygotes containing intron 1 with the corresponding deletion mutation, however, the *tva*^s/r3^, *tva*^s/r4^, *tva*^s/r5^ and *tva*^s/r6^ heterozygotes generate both the normal splicing of shorter *tva* forms and abnormal splicing of longer and shorter *tva* forms. Stars represent premature TGA stop codons identified in the alternative transcript.

Sequencing revealed that both the *tva*^r5^ and *tva*^r6^ alleles delete the CTCAC consensus sequence of the branch point signal^17^ (Fig. 1). These deletions include the A nucleotide, which is required for the first cleavage-ligation step of the splicing reaction^18^. Therefore, we hypothesized that the deletion mutations may disrupt splicing of the *tva* precursor mRNA (pre-mRNA) (Fig. 2A).

To determine whether these specific mutations would interfere with the process of *tva* pre-mRNA splicing, the full-length *tva* coding sequence from lives samples of defined origin and from DF-1 cells as a control were amplified by RT-PCR using primers crossing the entire coding sequence of the *tva* receptor gene (Fig. 2B). The cDNA products from the DF-1 cells and *tva*^s/s^ samples from lives were of the expected sizes corresponding to the longer and shorter *tva* isoforms.^13^ However, the cDNA products from the *tva*^r5/r5^ and *tva*^r6/r6^ homozygous samples from lives, as well as those from the *tva*^r3/r4^, *tva*^r3/r5^, *tva*^r3/r6^, *tva*^r4/r5^, *tva*^r4/r6^ and *tva*^r5/r6^ heterozygous samples, were longer by approximately 200 bp (Fig. 2C), similar to those of the *tva*^r3/r3^ and *tva*^r4/r4^ homozygous samples^14^. These longer sequences suggest that the first intron is retained (i.e., nucleotides 337 to 530, based on the published *tva*^s^ allele genomic sequence, GenBank accession number: AY531262.1).Interestingly, the products from *tva*^s/r3^, *tva*^s/r4^, *tva*^s/r5^ and *tva*^s/r6^ heterozygous samples from lives by RT-PCR have three transcripts. Except for one transcript was the same with the normal shorter *tva* isoforms, the another two transcripts were longer by approximately 200 bp compared with those of the longer and shorter *tva* isoforms, respectively (Fig. 2C).

In order to verify the retention of intron 1 in the transcripts of the *tva*^r5^ and *tva*^r6^ alleles, as well as to identify the heterozygotes, their RT-PCR products were then cloned into the T-vector for sequencing. Separate sequence analysis of PCR products revealed that the retention of the first intron in the RT-PCR products and a deletion of the corresponding sequence (CGCTCACCCC or CGCTCACCCCGCCCC) within the first intron in both the longer and shorter *tva* isoforms of *tva*^r5/r5^ and *tva*^r6/r6^ homozygotes, as well as the group of heterozygotes included *tva*^r3/r4^, *tva*^r3/r5^, *tva*^r3/r6^, *tva*^r4/r5^, *tva*^r4/r6^ and *tva*^r5/r6^ (Fig. 2D). Although these similar sequencing results were found in the *tva*^s/r3^, *tva*^s/r4^, *tva*^s/r5^ and *tva*^s/r6^ heterozygotes, the splicing of the shorter *tva* isoforms in these heterozygotes were normal (Fig. 2D). Therefore, the retention of intron 1 in both the longer and shorter *tva* isoforms of the *tva*^r5^ and *tva*^r6^ alleles were confirmed via alternative splicing, as well as identified in the heterozygotes. The position of the consensus sequence (CTCAC) of the putative branch point signal positioned at 23 nucleotides upstream of the 3′ splice signal, which is within the optimal distance from the intron-exon boundary^19^,^20^, suggesting that it is used in splicing out the first intron from the *tva* pre-mRNA and its deletion would abrogate the splicing event. Retention of intron 1 in the *tva*^r5^ allele changes the translational reading frame of both forms of the *tva* mRNAs immediately after the end of exon 1 and introduces a premature stop codons TGA in exon 4 or 5 of the longer or shorter mRNA, respectively (Fig. 2D). These two nonspliced mRNAs should be translated into proteins of 196 and 177 amino acids, respectively. However, the retention of the first intron in the *tva*^r6^ allele changes the translational reading frame of both forms of the *tva* mRNAs immediately after the end of exon 1 and introduces premature a stop codon TGA in exon 3 of both the longer and shorter mRNA, causing both nonspliced mRNAs to be translated into proteins of 167 amino acids (Fig. 2D). Therefore, we have demonstrated that intronic deletions comprising the deduced branch point signal within the first intron lead to inefficient splicing of *tva* mRNA.

### Construction of ASLV subgroup A reporter virus

**Figure 3:**
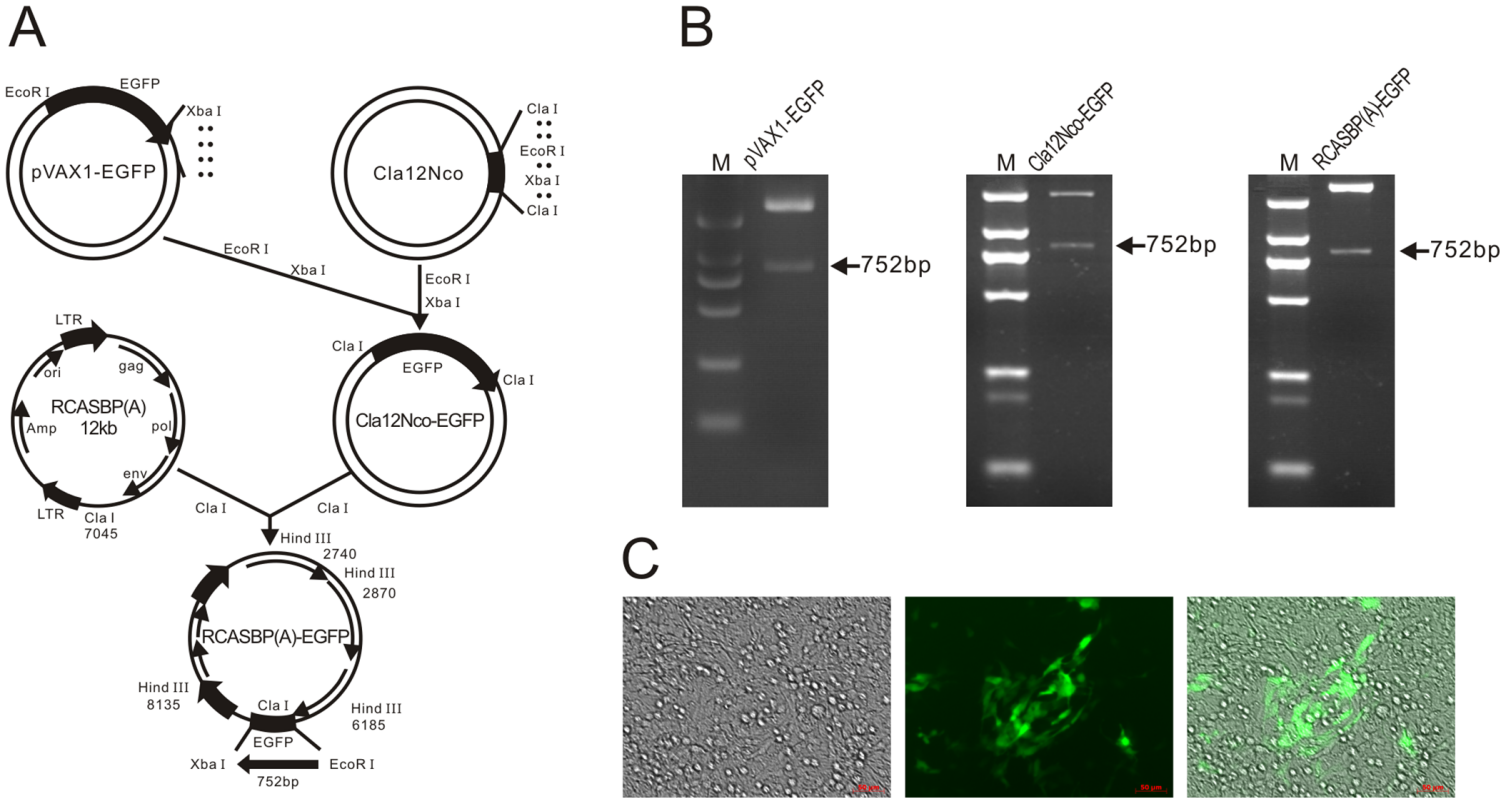
Construction of RCASBP(A)-EGFP. (A) Schematic representation of strategy for construction of RCASBP(A)-EGFP. Restriction sites used for construction of RCASBP(A)-EGFP are indicated. *EGFP*, with the size, is indicated. (B) Restriction enzyme digestion analysis of pVAX1-EGFP, Cla12Nco-EGFP and RCASBP(A)-EGFP vectors. (C) Fluorescence microscopy of transduced DF-I cells confirming *EGFP* marker gene expression.

To simplify the procedure and improve the quantitative assessment of ASLV-A infection, we constructed an expression vector RCASBP(A)-EGFP to produce the green fluorescent protein (GFP) reporter virus of subgroup A specificity based on the RCAS retrovirus vector^21^ (Fig. 3A). The *EGFP* gene was successfully cloned into the RCASBP(A) vector (Fig. 3B). The resulting replication-competent virus, RCASBP(A)-EGFP, was propagated in DF-1 cells and reached a titer of 10^6^ IU/mL. We then checked the subgroup specificity of the RCASBP(A)-EGFP virus by infection of chicken DF-1 cells and human 293 T cells. The proportion of GFP-positive cells in virus-inoculated DF-1 cells gradually increased over time, providing evidence for the replication and spread of virus (Fig. 3C). By comparison, less than 0.1% of inoculated as well as non-inoculated 293 T cells were GFP-positive, which may be attributed to the background auto-fluorescence (data not shown).

### Reduced susceptibility of chinese chickens to subgroup A ASLV

**Figure 4:**
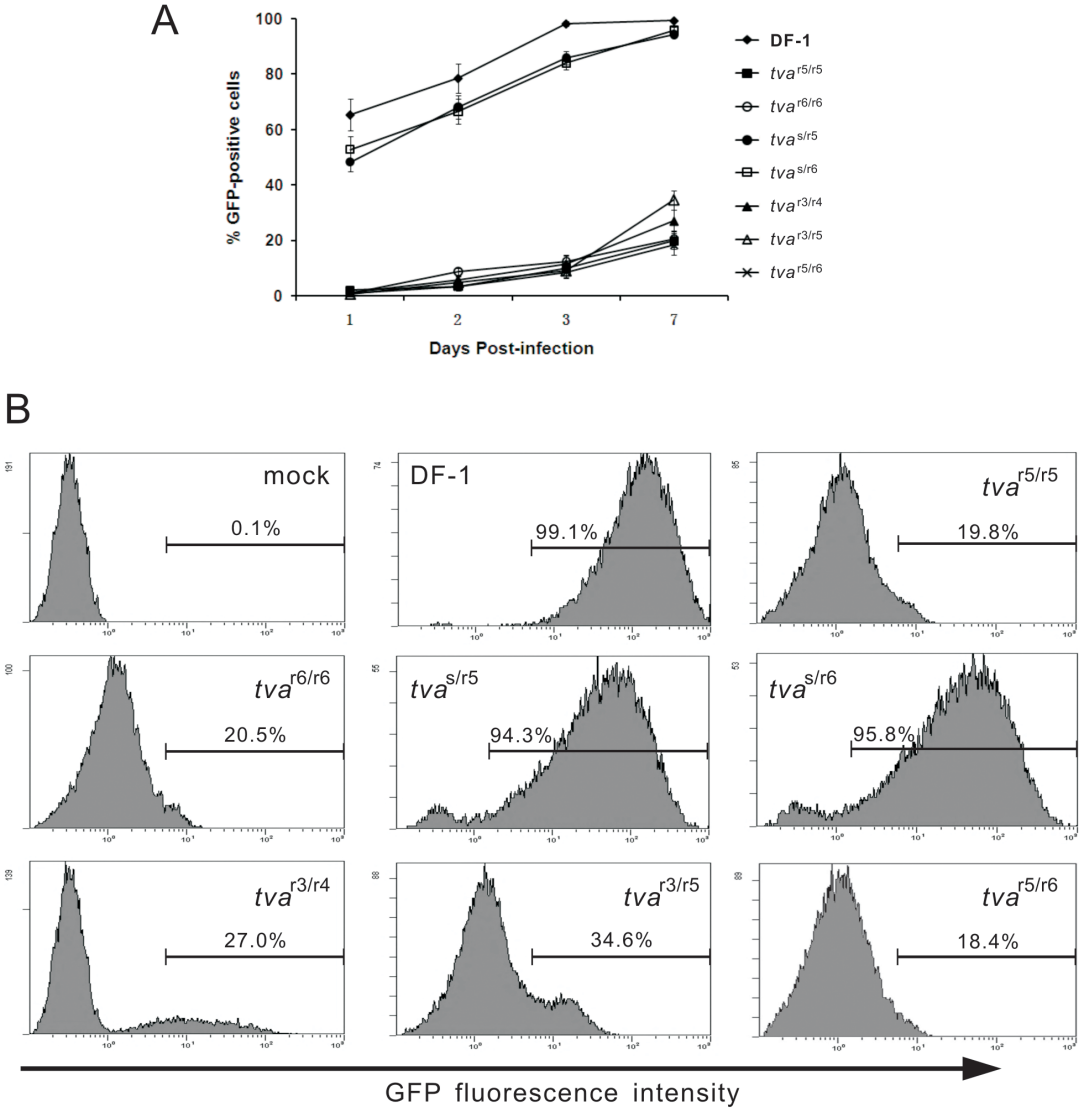
Time course of ASLV-A infection of DF-I cells and CEFs of defined origin. DF-I cells (*tva*^s/s^) and CEFs of defined origin were inoculated at a the multiplicity of infection of 10 with replication-competent ASLV encoding the EGFP reporter protein, RCASBP(A)-EGFP. (A) Proportions of GFP-positive cells were determined by FACS on indicated days post-infection. Results are means of data from three parallel dishes. (B) Representative FACS histograms of CEFs infected with RCASBP(A)-GFP at 7 days post-infection. The relative GFP fluorescence is plotted against the cell count, and the percentage of GFP-positive cells is indicated.

Since two independent intronic deletions that resulted in defective splicing of *tva* mRNA were identified, we hypothesized that these deletion mutations may confer their functional significance. To determine the effects of *tva*^r5^ and *tva*^r6^ alleles, as well as their heterozygous variants, on subgroup A ASLV susceptibility, CEFs of defined origin were infected with the RCASBP(A)-EGFP reporter virus, a replication-competent ASLV vector encoding EGFP, and the time course of infection was followed by quantitating the percentage of green fluorescent cells by flow cytometry over 7 subsequent days. DF-1 cells (*tva*^s/s^) are susceptible to subgroup A ASLV and thus were used as a positive control. As expected, the DF-1 cells was efficiently infected by RCASBP(A)-EGFP, with more than 60% of the cells being infected on day 1 post-infection, and virtually complete infection of cells was achieved by day 3 (Fig. 4A). However, a very different result was observed when the *tva*^r5/r5^ and *tva*^r6/r6^ CEFs were infected with RCASBP(A)-EGFP. Both the *tva*^r5/r5^ and *tva*^r6/r6^ CEFs were much less efficiently infected with RCASBP(A)-EGFP, with only 2.0% and 1.0% of the cells infected on day 1, respectively, and the virus spread very slowly, with only 19.8% and 20.5% of the cells infected by day 7, respectively (Fig. 4C). We also determined the susceptibility of *tva*^r3/r3^ and *tva*^r4/r4^ CEFs to subgroup A ASLV, and the results were consistent with those reported by Reinišová *et* *al.*^14^ (date not shown). In a separate experiment, quantitative differences in host susceptibility to ASLV-A were observed between heterozygotes which could be classified into two groups. The *tva*^s/r5^ and *tva*^s/r6^ CEFs were efficiently infected with RCASBP(A)-EGFP at a similar rate to that of DF-1 cells (*tva*^s/s^) (Fig. 4B), with 94.3% and 95.8% of the cells infected by day 7, respectively (Fig. 4C). However, the *tva*^r3/r4^, *tva*^r3/r5^ and *tva*^r5/r6^ CEFs were inefficiently infected by RCASBP(A)-EGFP (Fig. 3B), with only 27.0%, 34.6% and 18.4% of the cells being infected at 7 days post-infection, respectively (Fig. 4C). These results clearly indicated the inefficient infection and slow spread of the subgroup A ASLV in the *tva*^r5/r5^ and *tva*^r6/r6^ homozygous CEFs and the *tva*^r3/r4^, *tva*^r3/r5^ and *tva*^r5/r6^ heterozygous CEFs.

### Inefficient infection of chinese chickens by subgroup A ALV

In order to further determine the *in vivo* effects of the *tva*^r5^ and *tva*^r6^ alleles, as well as their heterozygous variants, commercial broilers randomly collected from lines 208, 419, 502 and 603 were inoculated with subgroup A ALV, and blood samples of chicks were tested for ALV-A infection. One-day-old chicks were inoculated with 0.2 mL of subgroup A ALV (2.4 S/P) into the abdominal cavity, and inoculated once again when they were 5 days old. At one month post-infection, the status of ALV-A infection was determined by RT-PCR of RNA extracted from whole blood samples. As expected, the *tva*^s/s^ birds were all positive for ALV-A. While the *tva*^s/r3^, *tva*^s/r4^, *tva*^s/r5^ and *tva*^s/r6^ birds were nearly all positive for ALV-A, in the cohort of 9, 5, 9 and 5 of the *tva*^s/r3^, *tva*^s/r4^, *tva*^s/r5^ and *tva*^s/r6^ chicks, respectively, all birds were positive for ALV-A, except for one *tva*^s/r5^ bird (Table 2). However, the susceptibility to subgroup A ALV of the homozygous *tva*^r3/r3^, *tva*^r4/r4^, *tva*^r5/r5^ and *tva*^r6/r6^ chicks and the heterozygous *tva*^r3/r4^, *tva*^r3/r5^, *tva*^r3/r6^, *tva*^r4/r5^, *tva*^r4/r6^ and *tva*^r5/r6^ chicks decreased. In the cohort of 32, 18, 15, 11, 6, 9, 5, 8, 6 and 17 of the *tva*^r3/r3^, *tva*^r4/r4^, *tva*^r5/r5^, *tva*^r6/r6^, *tva*^r3/r4^, *tva*^r3/r5^, *tva*^r3/r6^, *tva*^r4/r5^, *tva*^r4/r6^ and *tva*^r5/r6^ chicks, only 4, 1, 1 and 1 of the *tva*^r3/r3^, *tva*^r4/r4^, *tva*^r6/r6^, and *tva*^r5/r6^ chicks were positive for ALV-A (Table 2). These results were consistent with those of the *in vitro* experiment.

Taken together, these results clearly demonstrated that the deletion of the branch point signal within the first *tva* intron disrupt mRNA splicing of the *tva* receptor gene, which explains the decreased susceptibility of the homozygous *tva*^r5/r5^ and *tva*^r6/r6^ birds and the heterozygous *tva*^r3/r4^, *tva*^r3/r5^, *tva*^r3/r6^, *tva*^r4/r5^, *tva*^r4/r6^ and *tva*^r5/r6^ birds to infection by subgroup A ASLV.

## Discussion

In the present study, we described the identification of two similar intronic deletions encompassing the deduced branch point signal in the *tva* receptor gene, which were shown to significantly decrease the susceptibility of chickens to infection by ASLV-A. Furthermore, we identified two groups of heterozygotes based on their quantitative differences in host susceptibility to ASLV-A. This study is the first to report genetic defects in the *tva* receptor gene that account for a quantitative effect on ASLV-A susceptibility and pathogenesis in Chinese chickens. The altered susceptibility to infection by ASLV-A was observed both in cultured cells and in chicks challenged with subgroup A ALV.

Alternative splicing allows individual genes to produce two or more variant mRNAs, which in many cases encode functionally distinct proteins^19^,^22^,^23^. Indeed, two *tva* receptor gene splicing variants created by alternative splicing were identified previously^7^,^24^. Binding of more than one receptor, probably two, is needed for entry of virions via the Tva800 receptor that is encoded by the shorter *tva* forms, whereas binding of just one Tva950 receptor encoded by the longer *tva* forms is sufficient for successful entry^25^. Alternative splicing can be modulated by variation both in the *cis* genomic splicing signals and in the cellular pathways that regulate splicing^22^,^26^,^27^. In this study, we identified the alternative splicing of the *tva*^r5^ and *tva*^r6^ resistant alleles resulting from intronic deletions encompassing the deduced branch point signal, leading to the retention of the first intron and introduction of premature TGA stop codons in the longer and shorter *tva* forms (Fig. 2). Alternative splicing events generally create a premature termination codon that would cause the resulting mRNA to be degraded by nonsense-mediated mRNA decay^28^,^29^. Therefore, alternative splicing of mRNAs that changes the encoded proteins has profound functional effects^23^,^30^,^31^. Experimental analysis of distinct protein isoforms showed that alternative splicing regulates binding between proteins, between proteins and nucleic acids, as well as between proteins and membranes^23^,^31^,^32^,^33^. In most cases, the binding affinity is modulated, but the binding is not abolished completely^34^,^35^,^36^,^37^. The *tva*^r3^ and *tva*^r4^ alleles harbor an intronic deletion of the branch point and disrupt splicing of the *tva* receptor gene, leading to decreasing binding affinity between the Tva receptor and envelope glycoproteins of ASLV-A and display of the Tva receptor on the cell surface, and consequently reduced host susceptibility to subgroup A ASLV^14^. Given that the *tva*^r3^, *tva*^r4^, *tva*^r5^ and *tva*^r6^ alleles all contain a deletion of the same branch point signal, the same effects are also likely exerted by the *tva*^r5^ and *tva*^r6^ alleles in homozygotes, as well as the *tva*^r3/r4^, *tva*^r3/r5^, *tva*^r3/r6^, *tva*^r4/r5^, *tva*^r4/r6^ and *tva*^r5/r6^ resistant heterozygotes, as evidenced by their significantly decreased susceptibility to infection by ASLV-A. In contrast to resistant heterozygotes, the *tva*^s/r3^, *tva*^s/r4^, *tva*^s/r5^ and *tva*^s/r6^ susceptible heterozygotes may retain a receptor conformation that is at least partially suitable for binding of ASLV-A envelope glycoproteins and subsequent viral infection. A previously similar study were reported by Kučerová^16^, the deletion of W38 completely abrogates receptor activity and explains the resistance of chukar to the J subgroup of ALV; however, alleles with W38 replaced by G or E conferred susceptibility to the virus when overexpressed in the virus entry assay. Alternative splicing is not only important for normal cellular functions but also frequently is involved in disease pathogenesis^27^,^38^,^39^,^40^,^41^. Interestingly, published studies have identified intronic deletions causing exon skipping or intron retention in the human *LDLR* gene of patients with familial hypercholesterolemia^42^,^43^,^44^,^45^,^46^. Because the human *LDLR* gene is homologous to the *tva* receptor gene, such deletions as described above may be speculated as a common mechanism of mutagenesis within this gene family.

From the point of view of virus-host coevolution, it is tempting to speculate that the *tva*^r5^ and *tva*^r6^ alleles with decreased susceptibility to ASLV-A in Chinese chickens have been selected by pressure from subgroup A ASLV. Although eradication management strategies at the breeder level and high biosecurity level of flock production are used to control avian leukosis^47^,^48^, these conventional methods are not a practical means of completely eliminating the occurrence and spread of infection in developing countries^49^,^50^,^51^. The prevalence of ASLV in chicken populations may have imposed a strong selection pressure toward resistance or at least decreased susceptibility to ASLV infection. ASLV infection is known to cause a variety of neoplastic disease conditions and other production problems in affected flocks^52^. Under these conditions, a wide prevalence of resistance alleles in the ASLV receptor genes can be expected. To date, resistance alleles in the *tva*, *tvb* and *tvc* genes have been found in particular inbred lines of White Leghorns^11^,^12^,^13^,^14^,^15^. The resistance conferred by some alleles are caused by premature termination codons or frame shift mutations, such as *tva*^r2^, *tvb*^r^ and *tvc*^r11–13^, which do not encode any product that can carry out normal cellular functions, but their potentially detrimental impact on resistant birds is unknown. Given the presence of such counter-selection and rapid evolution of ASLV envelope glycoproteins, complete resistance to ASLV entry can appear but cannot prevail and be fixed in the chicken population. Hence, we suggest that genetic variations with modest effects on both ASLV entry and natural receptor functions provided a positive selective advantage in the chicken population. Selection of mutant Tva proteins that have lost the ability to function as an ASLV-A receptor while possibly retaining the“normal” Tva function may have provided the greatest selective advantage, resulting in fixing of the *tva*^r5^ and *tva*^r6^ loci in the germ lines of certain lines of chickens.

The *tva*^r3^, *tva*^r4^, *tva*^r5^ and *tva*^r6^ resistant alleles are prevalent in Chinese commercial broiler lines (Table 1), indicating that the potential for genetic improvement of resistance to ASLV-A is great and selective breeding for chickens genetically resistant to ASLV-A is feasible. Despite the potential benefits to be derived from breeding for enhanced resistance to ASLV-A, evaluating whether trade-offs between disease resistance and other economically important traits of chickens exist would be prudent. In fact, in a separate study we have evaluated the effects of genetic resistance to subgroup A ASLV on growth, feed efficiency and carcass traits using an F2 resource population by reciprocally crossing Huiyang Bearded chickens and fast-growing Chinese yellow broilers. The results demonstrated that genetic selection for *tva*^r3^, *tva*^r4^, *tva*^r5^ and *tva*^r6^ resistant loci can improve genetic resistance to subgroup A ASLV, but does not compromise production performance (data not shown). Selection for genetic resistance to subgroup B ASLV has been found to have no negative effect on laying performance in White Leghorn hens^53^. Furthermore, genetic selection for *tva*^r^ and *tvb*^r^ alleles can improve laying performance and growth rate, as well as greatly reduce lymphoid leukosis in White Leghorn hens^54^,^55^.

In conclusion, our study identified two novel genetic resistant alleles, *tva*^r5^ and *tva*^r6^, in Chinese commercial broilers. These two resistant alleles both harbor an internal intronic deletions comprising the deduced branch point signal within the first intron and leading to alternative splicing of the *tva* receptor gene, resulting in significantly decreased the susceptibility to subgroups A ASLV. We also demonstrated a difference in the susceptibility to subgroup A ASLV between *tva*^r3/r4^, *tva*^r3/r5^, *tva*^r3/r6^, *tva*^r4/r5^, *tva*^r4/r6^ and *tva*^r5/r6^ heterozygotes and *tva*^s/r3^, *tva*^s/r4^, *tva*^s/r5^ and *tva*^s/r6^ heterozygotes. This study provides valuable insight into mechanisms of genetic resistance to ASLV and retrovirus-host coevolution.

## Methods

### Ethics statement

The animal experiments were conducted in accordance with the guidelines of the Guangdong province on the review of welfare and ethics of laboratory animals approved by the Guangdong province administration office of laboratory animals (GPAOLA). All the animal procedures were approved by the Animal Care Committee of the College of Animal Science, South China Agricultural University, Guangzhou, China (approval ID: 201004152).

### Amplification and analysis of *tva* alleles from commercial broiler lines

Genomic DNA was prepared from blood samples of 22 commercial broiler lines using phenol-chloroform extraction. These commercial broiler lines were maintained at Guangdong Wen’s Food Group Co., Ltd. The sequence of the genomic region including exon 1, intron 1 and exon 2 of the *tva* gene was amplified using forward TVA1 primer 5′-GTTCAGCAGATCCTCATCTCCCG-3′ and reverse TVA2 primer 5′-GGCCATTGTGCGATCTAAGAGGG-3′. The PCR procedure was as follows: an initial denaturation at 95°C for 5 min, followed by 35 cycles of 94°C for 30 s, 67°C for 45 s and 72°C for 90 s, and a final extension of 72°C for 10 min with KOD-Plus Neo (Toyobo, Tokyo, Japan). In total, 712 birds from different commercial broiler lines were genotyped. The final PCR product with an expected length of 1308 bp was directly sequenced using an ABI 3730 sequencer (Applied Biosystems, Foster City, CA, USA).

### Splicing analysis by RT-PCR

Total RNAs from lives of commercial broiler lines 208, 419, 502 and 603 were isolated using TRIZOL reagent (Invitrogen, Carlsbad, CA, USA) according to the manufacturer’s instructions. cDNA was obtained by reverse transcription of 1 μg of total RNA with ReverTra Ace® qPCR RT Master Mix with gDNA Remover (Toyobo, Tokyo, Japan). The whole *tva* coding sequence was amplified using the forward TVA3 primer 5′- CCGGCATGGTGCGGTTGTTG-3′ containing the *tva* initiation codon and the reverse TVA4 primer 5′-AGCCAGGTTCCACGGTCAGC-3′, which is complementary to the 3′ untranslated region. The PCR conditions were as follows: an initial denaturation at 95°C for 5 min, followed by 30 cycles of 94°C for 30 s, 58°C for 30 s and 72°C for 50 s, and a final extension of 72°C for 5 min with KOD FX (Toyobo, Tokyo, Japan). To identify the transcripts of the *tva* alleles and their heterozygotes, the RT-PCR products were visualized by electrophoresis on 2% agarose gels. The purified RT-PCR products were ligated into the pMD19-T vector (TaKaRa, Dalian, China) and sequenced using an ABI 3730 sequencer (Applied Biosystems).

### Construction of ASLV subgroup A reporter vector and virus propagation

The *EGFP* gene was isolated from the pVAX1-EGFP vector (stored at our laboratory) as an *EcoR*I-*Xba*I fragment and cloned into the Cla12Nco adapter plasmid at the same restriction sites^21^,^56^. The resulting clones with the proper orientation of *EGFP* were isolated as *Cla*I fragments and subcloned into the corresponding site of the RCASBP(A) vector^21^, which was kindly obtained from Stephen H. Hughes (HIV Drug Resistance Program, National Cancer Institute, USA). The resulting expression construct was designated RCASBP(A)-EGFP. The RCASBP(A)-EGFP virus, transducing the *EGFP* reporter gene, was propagated by transfection of plasmid DNA containing the reporter vector into DF-1 cells, which are free of closely related endogenous retrovirus loci. Transfection was performed by using the X-tremeGENE 9 transfection reagent (Roche, Basel, Switzerland) according to the manufacturer’s instructions. Infection and virus spread were observed as an increasing proportion of GFP-positive cells, and virus stocks were harvested on day 9 post-transfection. The cell supernatants were cleared of debris by centrifugation at 2,000 × *g* for 10 min at 10°C, and aliquoted viral stocks were stored at −80°C. The virus titer was determined by terminal dilution and subsequent infection of DF-1 cells, reached 10^6^ IU/mL.

### Preparation of CEFs and cell culture

Primary CEFs were prepared from 9 to 11-day-old embryos from commercial broilers lines 208, 419, 502 and 603. The procedure was described previously^57^. The genotypes of CEFs were determined by direct sequencing as described above. All embryo fibroblasts, as well as the chicken permanent cell line DF-1^58^, were propagated in growth medium containing Dulbecco’s modified Eagle’s medium (DMEM) (Gibco/Invitrogen, Carlsbad, CA, USA) with 10% fetal bovine serum (Gibco/Invitrogen, Carlsbad, CA, USA), and penicillin/streptomycin (100 mg/ml each) at 37°C and 5% CO_2_.

### RCASBP(A)-EGFP virus spread assayed by fluorescence-activated cell sorting (FACS)

CEFs of defined origin were mock infected or infected by incubation with diluted RCASBP(A)-EGFP virus stock. Briefly, the CEFs were seeded in triplicate wells at a density of 5 × 10^4^ per well in a 24-well plate and inoculated with 5 × 10^5^ IU of RCASBP(A)-EGFP virus 24 h after seeding. The virus was applied in 0.25 mL medium for 1 h. The percentage of GFP-positive cells was quantitated by FACS using a Cytomics FC 500 analyzer (Beckman Coulter, Brea, CA, USA) on days 1, 2, 3 and 7 post-infection. For FACS analysis, the cells of three wells were trypsinized, washed in phosphate-buffered saline (PBS) and then analyzed.

### Animal experiment

The animal experiment design included two independent experiments with 100 birds each. Chicks randomly collected from commercial broilers lines 208, 419, 502 and 603 were randomly divided into four groups with 25 birds each. Chicks were maintained in four negatively-pressured biosecurity isolators under quarantine conditions and provided with water and commercial feed *ad libitum*. One-day-old chicks were inoculated with 2.4 S/P of avian leukosis virus subgroup A (ALV-A) strain GD08^49^, which was kindly provided by Weisheng Cao at South China Agricultural University, P. R. China, in 0.2 mL into the abdominal cavity. These chicks were inoculated once again at five days of age. A whole blood sample from each one-day-old chick was drawn from the wing vein and used for genomic DNA isolation by the phenol-chloroform method for genotyping as above described. To determine whether blood samples of chicks were positive for ASLV-A at one month post-infection, a whole blood sample from each bird was drawn for preparing total RNA using the TRIZOL reagent (Invitrogen, Carlsbad, CA, USA). The coding sequence of the env gene of ALV-A was amplified using the forward H5 primer 5′- GGATGAGGTGACTAAGAAAG-3′ and reverse H6 primer 5′- AGAGAAAGAGGGGTGTCTAAGGAGA-3′^59^. The PCR conditions were as follows: a reverse translation at 50°C for 30 min, then an initial denaturation at 94°C for 3 min, followed by 30 cycles of 94°C for 30 s, 56°C for 30 s and 72°C for 60 s, and a final extension of 72°C for 5 min with PrimeScript® One Step RT-PCR Kit Ver. 2 (TaKaRa, Dalian, China). To identify the infection status of ALV-A, the PCR products were visualized by electrophoresis on 2% agarose gels.

## Author Contributions

W.G.C., Y.L. and Q.M.X. conceived and designed the project. W.G.C., Y.L. and H.X.L. performed the experiments. W.G.C., H.X.L. and S.C. analyzed the data. W.G.C. and H.M.Z. wrote the paper. D.M.S., H.M.Z. and F.C. contributed the materials. H.M.Z. and Q.M.X. carried out the principal investigator. W.G.C. and Y.L. contributed equally to the work. All of the authors read and approved the final manuscript.

## Figures and Tables

**Table 1:** Genotypic frequency of *tva*^mut502–516^ multiple allele^1^ in Chinese commercial broiler lines

Line	No.	*tva*^mut502–516^ genotype 2														
		*tva*s/s	*tva*s/r3	*tva*s/r4	*tva*s/r5	*tva*s/r6	*tva*r3/r3	*tva*r4/r4	*tva*r5/r5	*tva*r6/r6	*tva*r3/r4	*tva*r3/r5	*tva*r3/r6	*tva*r4/r5	*tva*r4/r6	*tva*r5/r6
202	44	0	0	0.12	0	0	0.07	0.52	0	0	0.02	0.11	0	0.11	0	0.05
203	29	0.31	0	0	0	0	0.10	0.41	0	0	0	0.14	0	0.03	0	0
204	30	0.23	0	0	0	0	0.17	0.27	0.03	0	0.13	0.10	0	0.07	0	0
205	30	0.57	0	0	0	0	0.17	0	0.13	0	0	0	0	0.10	0	0.03
207	29	0	0	0	0	0.08	0.03	0	0.26	0.10	0	0.03	0	0	0	0.41
208	27	0.22	0	0	0	0	0.19	0.11	0.37	0	0.04	0.04	0	0.04	0	0
209	28	0.18	0	0	0	0	0.21	0.00	0.43	0	0.07	0.04	0	0.04	0	0.04
301	41	0.20	0	0	0	0	0.20	0.02	0.34	0	0.02	0.17	0	0.02	0	0.02
306	31	0	0	0	0.13	0	0.16	0	0.55	0	0	0.10	0.03	0	0	0.03
406	26	0.27	0.04	0	0	0	0.31	0.08	0	0	0.12	0.08	0	0.08	0.04	0
407	40	0.10	0.05	0.05	0	0	0	0.05	0.35	0	0.05	0.05	0	0.30	0	0
408	30	0.07	0	0	0.07	0	0.10	0.27	0	0	0.27	0.17	0	0.07	0	0
411	27	0.04	0	0	0	0	0.15	0.30	0.11	0.04	0.22	0.07	0.04	0	0.04	0
413	29	0.38	0.03	0.03	0	0	0.28	0	0	0	0.21	0.03	0	0.03	0	0
417	42	0.14	0	0	0	0	0.14	0.29	0.05	0	0.19	0.05	0	0.14	0	0
418	28	0.14	0.04	0	0	0	0.25	0.07	0.04	0	0.18	0.25	0	0.04	0	0
419	46	0.13	0	0	0	0	0.04	0.33	0.04	0.02	0.04	0.17	0	0.04	0.04	0.13
501	29	0.17	0	0	0	0	0.17	0.07	0	0	0.34	0.17	0	0.07	0	0
502	28	0.21	0	0	0.04	0.04	0.21	0.11	0	0	0.04	0.21	0	0.04	0.11	0
505	30	0.13	0	0	0	0	0.07	0.07	0.47	0	0	0.07	0	0.20	0	0
511	38	0.32	0	0	0	0	0.11	0.11	0.16	0	0	0.05	0	0.16	0	0.11
603	30	0.20	0.03	0.03	0.07	0	0.13	0.20	0.03	0	0.07	0.10	0.07	0.03	0	0.03

^1^*tva*^mut502–516^ = multiple allele of *tva*^r3^, *tva*^r4^, *tva*^r5^ and *tva*^r6^ at the *tva* locus.

^2^*tva*^s/s^ = susceptible homozygote of *tva* receptor gene; *tva*^s/r3^ = heterozygote of deleted ACCCCGCCCC; *tva*^s/r4^ = heterozygote of deleted ACCCC; *tva*^s/r5^ = heterozygote of deleted CGCTCACCCC; *tva*^s/r6^ = heterozygote of deleted CGCTCACCCCGCCCC; *tva*^r3/r3^ = homozygote of deleted ACCCCGCCCC; *tva*^r4/r4^ = homozygote of deleted ACCCC; *tva*^r5/r5^ = homozygote of deleted CGCTCACCCC; *tva*^r6/r6^ = homozygote of deleted CGCTCACCCCGCCCC; *tva*^r3/r4^ = heterozygote of deleted ACCCCGCCCC and ACCCC; *tva*^r3/r5^ = heterozygote of deleted ACCCCGCCCC and CGCTCACCCC; *tva*^r3/r6^ = heterozygote of deleted ACCCCGCCCC and CGCTCACCCCGCCCC; *tva*^r4/r5^ = heterozygote of deleted ACCCC and CGCTCACCCC; *tva*^r4/r6^ = heterozygote of deleted ACCCC and CGCTCACCCCGCCCC; and *tva*^r5/r6^ = heterozygote of deleted CGCTCACCCC and CGCTCACCCCGCCCC.

**Table 2:** Incidence of RT-PCR positivity for avian leukosis virus subgroup A at one month post-infection in chicks with different genotypes of the *tva*^mut502–516^ multiple allele^1^

*tva*^s/s2^	*tva*s/r3	*tva*s/r4	*tva*s/r5	*tva*s/r6	*tva*r3/r3	*tva*r4/r4	*tva*r5/r5	*tva*r6/r6	*tva*r3/r4	*tva*r3/r5	*tva*r3/r6	*tva*r4/r5	*tva*r4/r6	*tva*r5/r6
45/45	9/9	5/5	8/9	5/5	4/32	1/18	0/15	1/11	0/6	0/9	0/5	0/8	0/6	1/17

^1^*tva*^mut502–516^ = multiple allele of *tva*^r3^, *tva*^r4^, *tva*^r5^ and *tva*^r6^ at the *tva* locus.

^2^*tva*^s/s^ = susceptible homozygote of *tva* receptor gene; *tva*^s/r3^ = heterozygote of deleted ACCCCGCCCC; *tva*^s/r4^ = heterozygote of deleted ACCCC; *tva*^s/r5^ = heterozygote of deleted CGCTCACCCC; *tva*^s/r6^ = heterozygote of deleted CGCTCACCCCGCCCC; *tva*^r3/r3^ = homozygote of deleted ACCCCGCCCC; *tva*^r4/r4^ = homozygote of deleted ACCCC; *tva*^r5/r5^ = homozygote of deleted CGCTCACCCC; *tva*^r6/r6^ = homozygote of deleted CGCTCACCCCGCCCC; *tva*^r3/r4^ = heterozygote of deleted ACCCCGCCCC and ACCCC; *tva*^r3/r5^ = heterozygote of deleted ACCCCGCCCC and CGCTCACCCC; *tva*^r3/r6^ = heterozygote of deleted ACCCCGCCCC and CGCTCACCCCGCCCC; *tva*^r4/r5^ = heterozygote of deleted ACCCC and CGCTCACCCC; *tva*^r4/r6^ = heterozygote of deleted ACCCC and CGCTCACCCCGCCCC; and *tva*^r5/r6^ = heterozygote of deleted CGCTCACCCC and CGCTCACCCCGCCCC.
